# Physical frailty and fitness of older driver

**Published:** 2017-06-30

**Authors:** Maria Helena Lenardt, Clovis Cechinel, Maria Angelica Binotto, Nathalia Hammerschmidt Kolb Carneiro, Tânia Maria Lourenço

**Affiliations:** 1, Federal University of Paraná, Curitiba, Brazil, Curitiba. Brazil.

**Keywords:** Frail elderly, physical fitness, aged, quality of life, automobile driving, Brazil

## Abstract

**Aim:**

to analyze the association between physical frailty and the results of fitness capacity exams for driving vehicles among elder Brazilians.

**Methods:**

this is a cross sectional study, performed in traffic medicine clinics of the city of Curitiba (Brazil). The data was collected through the physical frailty tests, the use of a structured questionnaire, and searches on the records of the Brazilian National Register of Qualified Drivers.To analyze the information, the authors used descriptive statistics and non-parametrical tests.

**Results:**

One hundred seventy two elderly people, of whom 56.4% pre-fragile and 43.6% non-fragile. 25.0% were considered fit for driving, 68.6% were considered fit, but with some restrictions, and 6.4% were placed as temporarily unfit for driving. There was no association between frailty condition and the final results for driving fitness (*p*= 0.8934). Physical frailty was significantly associated to the restrictions observed for those who were fit under restrictions (*p*= 0.0313), according to the weekly amount of kilometers traveled (*p*= 0.0222), and to car accidents occurred after the age of 60 (*p*= 0.0165).

**Conclusion:**

Physical frailty was significantly associated to the restrictions related to driving, reason to which makes important to manage frailty over this group of drivers. However, no association observed between physical frailty and the final result for driving vehicles.

## Resumen

**Objetivo:**

Analizar la asociación entre la fragilidad física y los resultados del examen de aptitud para conducir un vehículo, en ancianos brasileños.

**Métodos:**

Estudio cuantitativo transversal, realizado en las clínicas de medicina de tráfico de Curitiba/PR (Brasil). Se recopilaron datos mediante pruebas de evaluación de la fragilidad física, aplicación de cuestionario estructurado y búsqueda en el formulario de Registro Nacional de Conductores Habilitados. Se utilizaron pruebas descriptivas y no paramétricas.

**Resultados:**

Ciento setenta y dos individuos de edad avanzada participaron en el estudio, de los cuales 56.4% fueron ancianos pre-frágiles y 43.6% no frágiles. Para conducir estaban aptos el 25.0% de los ancianos, 68.6% aptos con restricción y 6.4% no aptos temporarios. No hubo asociación entre la condición de fragilidad y los resultados finales para aptitud vehicular (*p*= 0.8934). La fragilidad física estuvo significativamente asociada a las restricciones impuestas para aptos con restricciones (*p*= 0.0313), al número de kilómetros conducidos semanalmente (*p*= 0.0222) y a los accidentes ocurridos después de los 60 años (*p*= 0.0165).

**Conclusiones:**

La fragilidad física estuvo significativamente asociada a las restricciones impuestas para aptos con restricciones, razón por la cual, es importante la gestión de la fragilidad en este grupo de conductores. Sin embargo no se observó una asociación entre la fragilidad física y el resultado final para conducir un vehículo.

## Introduction

The elderly population in Brazil, presented in the National Survey by Household Sampling in 2015, showed 29,374,000, equivalent to 14.3% of the general population ^1^, a higher proportion than in 2010, which represented 11.7% ^2^ . According to the same estimate, Paraná was the state with the 9th largest population of elderly in the country, formed by 1,637,000 individuals, which corresponds to 14.6% of the population ^1^.

The Statistical Yearbook of 2015 of the Paraná Transit Department (DETRAN) showed that 23.28% of drivers in the state are over 55 years old, corresponding to 1,237,471, with a higher number of men (950,886) compared to women (286,585) ^3^.

Aging and safe driving have a complex interface, which demands the integration of cognitive, sensorial, and motor skills. These skills can change and/or deteriorate over the years, and as consequence, compromise the ability to drive ^4^. The functionality, the age, and the health status influence driving performance of older drivers, which makes important to comprehend the process to better explore safe driving strategies ^5^. 

The Brazilian National Traffic Code does not impose a maximum age for drivers. The decision is based on medical inspection, where the physician evaluates the physical and mental conditions of drivers, as well as their skills, to drive ^6^. The process to renew the Brazilian National Driving License does not take into consideration the peculiarities of aging, being the same testing used in adults and young drivers. The only specificity for the older age group, present in the resolution #007/98, of the Brazilian National Traffic Council, is the demand to reduce from five to three years the interval for the medical inspection procedures necessary to renew the driving license for people above 65 years old ^7^. 

Among the many health issues, physical frailty is an important focus for investigation, due to its increased prevalence as people get older. Physical frailty is considered "a medical syndrome with multiple causes and contributions/determinants, in which is characterized by the reduction of strength, resistance, and reduction of physiological functioning that will increase the vulnerability of the individual, higher dependency, and/or death"^8^. 

The frailty phenotype is focused in the biological perspective of the syndrome, and it is composed by five measureable components, which includes the reduction of walking speed and handgrip strength, non-intentional weight loss, low level of physical activity, and self-reported fatigue/exhaustion ^9^. From these five components, the elder that present three or more of these characteristics is considered fragile; the ones that present one or two is placed in a previous stage to the syndrome (pre-frailty); and the ones that report none of the components mentioned is labeled as non-fragile. 

Studies that analyze elders in different contexts, such as traffic medicine clinics, generate a wider evaluation of this age group, and a deeper understanding of physical frailty syndrome, subsidizing the implementation of public policies to a safer driving practice.

In view of the above, the study aimed to analyze the association between physical frailty and results of the physical fitness test for vehicular direction in Brazilian elderly.

## Materials and Methods

This is a cross sectional quantitative study, in which the sample was built between January 31^st^ and July 31^st^ 2015. The study took place at the traffic medicine clinics in the city of Curitiba (Brazil) where driving abilities are evaluated. The criteria of inclusion of clinics were: being accredited to perform driving ability evaluation; having an appropriate physical space to perform such tests. 

During project designing, it was identified 54 regularly accredited clinics, which were put in sequence to be visited, chosen in a simple random sample. The clinics were classified with a letter and crescent numbers, from C1 to C54, and in this sequence they were evaluated according to the criteria of inclusion. 

Considering the lack of data related to the number of elders served by clinic, and the impartial and equitable distribution of elders by the traffic department, it was decided to establish a standard amount of interviewees (35 elders) to be interviewed in each clinic until the last day of data collection. In the last clinic, due to the end of this period, only 32 elders composed the sample. 

The criteria for inclusion of elders were: being 60 years old or older; being scheduled for the driving license tests; being able to answer the questionnaire. The criteria for exclusion were: to present illnesses and/or physical symptoms that, by any means, could impede to answer the questionnaire and to perform the tests. 

The elderly were invited to participate in the research and by signing the informed consent form; the data was collected in three stages: (1) use of the structured questionnaire; (2) tests to evaluate the physical frailty syndrome; (3) search for information in the Brazilian National Registry of Qualified Drivers. The structured questionnaire was designed specifically for the present study and it has questions related to driving skills. The variables of interest were: age, gender, type of driving license (first license or renewal), experience on driving, characteristics of driving performance (if it is mostly at night or during daylight, if travels only inside the neighborhood or goes frequently to downtown), used type of transmission (manual or automatic), average traveled kilometers per week, and car accidents after the age of 60. 

Physical frailty was evaluated through the components of Fried phenotype ^9^. Handgrip strength was measured with a hydraulic dynamometer, by Jamar^®^. The elders that were considered fragile in this component were the ones found in the lowest quintile, after adjustments for gender and body mass index.

To evaluate the walking speed, it was counted the seconds the participant took to go through a straight line under regular walking speed for 4.6 meters. After adjustments for gender and height, the elders that presented the results on the lowest quintile were considered fragile for this component.

The non-intentional weight loss was observed through two questions: Have you lost weight in the past 12 months? How much? It was considered fragile the ones who reported weight loss ≥4.5 kg in the past 12 months.

To evaluate the component fatigue/exhaustion, it was used the items 7 and 20 of the Scale of the Right for Epidemiological Studies - Depression (CES-D) (http://counsellingresource.com/quizzes/depression-testing/cesd/). The answers were categorized from 0 to 3, according to their frequency. An answer 2 or 3 to any of these questions placed the elder as fragile in this component.

The investigation of the level of physical activity was performed using the Minnesota Leisure Activity Questionnaire, validated in Brazilian elders ^10^. After adjustments for gender, the individuals on the lowest quintile were considered fragile in this component. 

The methods used to evaluate the physical fragility was proposed by Fried et al., but the cut-off points used in the present study were defined from the results of the sample.

In the Brazilian National Registry of Qualified Drivers there was a search for the final results of elders' evaluation in fitness test, and the values of handgrip strength collected by traffic physicians. The examination performed by the physician involves analysis, general physical examination, and specific exams that encompass, ophthalmological, otorhinolaryngological, cardiorespiratory, neurological and locomotor evaluation, and no direction examination is performed at this time. The result was presented according to the resolution 425, from November 27^th^ 2012, of the Brazilian National Traffic Council, in a scale of parameters labeled as "fit", "fit with restrictions", "temporarily unfit", and "unfit" ^6^. In the result "fit with restrictions", the physician describes the observed restrictions.

The data was organized and analyzed through the softwares Microsoft Excel^®^ 2015, and the Statistical Package for Social Sciences (SPSS), version 20.0. Descriptive statistics and non-parametric tests were used (chi-square and Cochan) to evaluate the association between interest variables and physical frailty. It was considered *p* ≤0.05 as for significant statistical evidence. 

This study was approved by the Ethics Committee for Researches in Human Beings, from the Health Sciences Department of Paraná Federal University (Brazil), under protocol CAAE 34689914.8.0000.0102; approval CEP/SD 833460. The ethical principles for volunteer and consented participation of each individual/clinic were observed, according to present regulations in Brazil, such as the Resolution #466 of the Brazilian National Health Council, from December 12^th^ 2012, which is based on the 1975 Helsinki Declaration and its further reviews. 

## Results 

The sample is composed by 172 elders, with an average age of 67.7 ±6.6 years old, being 120 (70.7%) men. No elder was evaluated and categorized as fragile; the pre-fragile were 97 (56.4%) individuals, and the non-fragile were 75 (43.6%) elders. The final result of the driving test performed by a traffic physician showed that 25.0% of these elders were considered fit, 66.6% of them were set as fit with restrictions, and 6.4% were seen as temporarily unfit. 

As seen on Table 1, the majority of the exams performed (169 tests, or 97.3% of the total) were aimed to renew the Brazilian National Driving License, which from those, more than 70% of the interviewees have more than 36 years of driving experience. When crossing the data from the Brazilian National Driving License records and the frailty condition, it was observed a significant association between frailty and the weekly traveled distance, in kilometers (*p*= 0.0222), and the number of accidents (*p*= 0.0165). There was no statistical significance among the characteristics related to driving (day/night, downtown/neighborhood, highways) and physical frailty.

**Table 1:** Association between physical frailty condition and the characteristics related to driving in elderly. Curitiba, Brazil, 2015

Variables	Pre-fragile (%)	Non-fragile (%)	Total	*p-value*
Type of Driving License				
Renewal	96(98.97)	73(97.33)	169	0.4173*
First License	1(1.03)	2(2.67)	3	
Driving Experience				
≥0 to<18	7(7.22)	8(10,67)	15	0.5783**
≥18 to<36	19(19.59)	13(17.33)	32	
≥36 to<54	64(65.98)	45(60)	109	
≥54 to<72	7(7.22)	9(12)	16	
Type of Transmission				
Manual	69(71.13)	55 (73.33)	124	0.9180*
Automatic	23(23.71)	17(22.67)	40	
Manual/automatic	5(5.15)	3(4)	8	
Weekly traveled distance in km				
≥0 to <50	16(16.49)	25(33.33)	41	0.0222**
≥50 to <250	60(61.86)	30(40)	90	
≥250 to <500	12(12.37)	13(17.33)	25	
≥500	9(9.28)	7(9.33)	16	
Accident				
Yes	9(9.28)	1(1.33)	10	0.0165*
No	88(90.72)	74(98.67)	162	
Night driving				
Yes	72(7.23)	53(70.67)	125	0.7035**
No	25(2.77)	21(28)	46	
Daylight driving				
Yes	96(98.97)	73(97.33)	169	0.8476*
No	1(1.03)	1(1.33)	2	
Downtown driving				
Yes	91(93.81)	64(85.33)	155	0.1030**
No	6(6.19)	10(13.33)	16	
Neighborhood driving				
Yes	95(97.94)	70(93.33)	165	0.2402*
No	2(2.06)	4(5.33)	6	
Highway driving				
Yes	73(75.26)	63(84)	136	0.1127**
No	24(24.74)	11(14.67)	35	

*Cochran Test -G Test, *p* ≤0.05

**Chi-square test, *p* ≤0.05.

On Table 2 is possible to observe the association between physical frailty and the results achieved by the elders in fitness test in the traffic medicine clinics. There was no association between the classification of the elder according to the Fried frailty phenotype and the final results in driving fitness (*p*= 0.8934).

**Table 2:** Association between physical frailty and final results of driving tests in traffic medicine clinics.Curitiba, Brazil, 2015

Final result on the physical and mental fitness test	Pre-fragile	Non-fragile	Total	*p-value*
Fit with restriction	65(67.01)	53(70.67)	118	0.8934
Temporarily unfit	6(6.19)	5(6.66)	11	
Fit	26(26.8)	17(22.67)	43	
Total	97(100)	75(100)	172	

Cochran Test - G Test, *p* ≤0.05.

In Table 3 there is the statistical association between physical frailty and the restrictions imposed to the candidates considered fit, but with restrictions (*p*= 0.0313). The use of contact lenses was the most prevailing restriction used (n= 32; 42.66%) among non-fragile.

**Table 3:** Association between frailty groups and the driving test result fit with restrictions. Curitiba, Brazil, 2015

Restrictions in driving license^*^	Pre-fragile	Non-fragile	Total	*p-value*
Use of corrective lenses	26 (26.8)	32 (42.66)	58	0.0313
Unauthorized category	24(24.8)	8 (10.67)	32	
Driving license validity for 2 years	2 (2.06)	4 (5.33)	6	
Unauthorized category, monocular vision capacity, and driving license validity for 2 years	1 (1.03)	0 (0)	1	
Use of corrective lenses and hearing aids	0 (0)	1 (1.33)	1	
Unauthorized category and use of lenses	11 (11.34)	6 (8)	17	
Unauthorized category and use of hearing aids	1 (1.03)	0 (0)	1	
Use of corrective lenses and driving license validity for 2 years Other restrictions**	0(0) 1 (1.03)	1 (1.33) 0 (0)	1 1	

*The test was performed considering only the fit with restrictions (n= 118);

**Other restrictions include: prohibited to drive in highways and fast lanes, prohibited to drive after sunset, driving license validity shortened to 2 years, use of lenses, and category downgrade

Cochran Test - G Test, *p* ≤0.05

## Discussion

From the evaluation of candidates to renew their Brazilian National Driving License, there were no fragile elders, and this information differs from the majority of studies, specially those that study infinite populations, including ill elders that have some compromised functional abilities and are considered fragile ^9^ ^,^ ^11^.Elders who wish to have a driving license are independent and autonomous.

Despite it was not observed the presence of fragile elders, the prevalence of pre-frailty was 56.4%, a finding that was above the ones reported in international researches, both in the United States of America ^11^ and Taiwan ^12^, scoring 40.0% and 45.5%, respectively. Similar percentage results that match this present study was found in countries of Latin America, such as Peru ^13^ (47.3%), and Colombia ^14^ (53.0%), which involved elders from local communities. The Brazilian studies of FIBRA (Frailty in Brazilian Elders, in Portuguese), pre-frailty varied between 47.7% in Ivoti, and 55.5% in Parnaíba ^15^. 

It is worrisome the high frequency of pre-fragile elder drivers, once it is impossible to predict when the evolution of a significant functional decline will occur, which will compromise safe driving for the conductor and for the society. Therefore, the identification of fragile and pre-fragile elders during fitness test for driving licensing is fundamental, and above all, the guidance of these elders to learn how to manage frailty under the responsibility of geriatric and gerontology health care. The management of frailty is comprehended under four multi-professional character interventions: the performance of physical exercises (resistance and aerobics), caloric and protein support, use of vitamin D, and reduction of polypharmacy ^8^.

There was a significant association between the report of traffic accidents and physical frailty after the age of 60. This result indicates that it is necessary to study the development of clinical prediction that is sensible and specific to detect the risk of accidents among elder drivers, mainly in those seen as fragile and pre-fragile.

The outcomes of traffic accidents involving elders can be different when comparing the age groups. The Insurance Commission of Western Australia - Motor Injury division - has compared two subgroups of drivers (ages between 45 and 64, and above 65 years old), and has found significant differences in regards to injuries. The youngest subgroup was more prone for injuries in the cervical region (30.6%), when compared to the result of the oldest group (12.1%), while the oldest group was more disposed to chest injuries (30.9%, against 18.5% of the youngest ones). Yet, the research demonstrates may not have the pulmonary capacity to recover from such injuries, being more inclined from dying from such damages^2^.

In regards to the driving habits of elders, it was found that 26.7% avoid driving at night, and 20.4% avoid highways and fast lanes. This growing self-regulation in night driving and highways was demonstrated in a study that characterized the drivers according to gender and age groups: 55-64 years old, 65-74 years old, and 75 years old or above. The increase in age was indirectly proportional to night driving, with a decrease in frequency of 30.4% among the youngest ones, 46.3% in the intermediary group, and 56.0% in the ones above 75 years old. The same occurred with highway driving: 18.8% of the youngest, and 32.9% of the oldest avoided driving in highways. When observing through gender, 34.9% of women avoided driving at night, and 24.4% avoided highways, while for men these numbers were 20.7% and 13.8%, respectively ^16^.

A study performed by the US Right for Disease Control (CDC) matches the results previously mentioned while showing that elders tend to modify their driving habits. As a result, it was found that 82% of the drivers who are 75 years old or more mentioned they limited their night driving travels, as well as due to weather or long distances. However, only 15% of the elders reported they limit their driving travels due to medical reasons, and none mentioned any cognitive impairment ^17^.

Elder drivers are conscious of their risks and use compensatory measures, avoiding dangerous driving conditions (such as heavy traffic or risky weather conditions), prefer taking known routes, and precautious driving techniques ^18^.

There was a statistic association between the restrictions in driving licenses and physical frailty of elders. The most frequent was the use of corrective lenses, and the unauthorized driving category statement. Comparing the pre-fragile elders to the non-fragile ones, the mandatory use of corrective lenses occurred less frequently among the pre-fragile. It is seen that many restrictions imposed to drivers are not related directly to the frailty phenotype, and are fundamental to driving, such as the sight.

In regards to the driving categories, it is noted the number of elders with unauthorized driving categories. It was seen that the most unauthorized category was "C", followed by category "A". The category "A" is formed by all motor and electrical engine vehicles of two or three wheels, with or without a side car; category "C" comprehends all motor and electrical engine vehicles used for cargo transportation, registered as with minimum load capacity of total gross weight of 6 tons and total gross weight of the vehicle cannot surpass 3.5 tons^19^. 

Candidates for driving vehicles under categories "C", "D", and "E" must demonstrate handgrip strength equal or above 30 kgf in each hand ^6^.From the 32 elders characterized under unauthorized category, half of them did not have an evaluated handgrip strength of 30 kgf, while the equipment in traffic medicine clinics found that only 31.25% of them failed the test, due to the reading interval of their dynamometers, which is 10 kgf. 

The minimum necessary dynamometer reading for the candidates to categories "A" and "B" must be equal or above 20 kgf ^6^. In the admeasurement performed in the evaluation of frailty, eight candidates presented handgrip below 20 kgf, however, none of them was considered unfit for driving. Among those, 25% were considered fit without restrictions, 50% were fit with visual restriction, and 25% had unauthorized categories, a fact that could lead to unsafe driving situations. More precise instruments could assist to show the candidate that the handgrip is near minimum standard, and to guide to activities that can improve muscular hand strength.

The existence of a relationship between physical frailty and the fit with restrictions demonstrates the necessity of an effective management of frailty, which should start at least during the evaluation for fitness test in order to keep the specific abilities for driving and ideal driving performance. The restriction to driving can lead to decrease quality of life, and at the same time it can increase senescence ^20^. Despite there was no association between physical frailty and the result of driving license, it was possible to note the association between frailty and the restriction imposed in the license. 

The lack of a passing score to walking speed, level of physical activity, and handgrip strength present in the frailty phenotype, as well as the presence of self-reported components, such as fatigue/exhaustion, level of physical activity, and unintentional weight loss can influence the classification of the elders.

Other limitations are related to the possibility of interviewees omitting data, during the physical frailty evaluation, and the transversal design of the study, which does not permit the complete understanding of some cause-and-effect relationships among the variables.

## Conclusion

Physical frailty was significantly associated to the restrictions related to driving, however, no association observed between physical frailty and the final result for driving vehicles, as well as an expressive percentage value of pre-fragile elders. This information demonstrates the importance of interventions in this stage, having higher possibility and effectiveness to interrupt and/or regress the condition, and as a consequence, the maintenance of a safe driving for a longer period. 
