# Supplementary material for: Physical frailty and fitness of older driver
Source: Colomb Med (Cali). 2017 Jun 30;48(2):41–6. (PMC5625565)
Supplement: Translation

# Translation: Fragilidad física en ancianos y aptitud para dirigir un vehículo

## Introducción

La población de edad avanzada en Brasil, que participó en la Encuesta Nacional por Muestra de los hogares en 2015, fue de 29,374,000, equivalente al 14.3% de la población general ^1^, una proporción mayor que la observada en 2010 que representó el 11.7% ^2^. Paraná fue el estado con la novena mayor población de personas mayores en el país, equivalente a 1,637,000 de personas, lo que correspondió al 14.6% de la población ^1^.

El Anuario Estadístico 2015 de la Dirección General de Tráfico de Paraná (DMV) mostró que el 23.3% de los conductores con licencia son mayores de 55 años, lo que corresponde a una población de 1,237,471, en su mayoría hombres (950,886) ^3^.

El envejecimiento y la conducción vehicular segura tienen una interface compleja, que exige la integración de funciones cognitivas, sensoriales y motoras. Estas funciones pueden cambiar y/o deteriorarse con el envejecimiento y consecuentemente, perjudicar el acto de conducir ^4^. La funcionalidad, la edad y el estado de salud influyen en el acto de conducir en la población adulta, por lo que es importante comprender este proceso para desarrollar estrategias de conducción segura ^5^. 

El Código de Tráfico Brasileño no determina la edad máxima para conducir vehículos automotores. La decisión se basa en el examen médico pericial, en el que se evalúan las condiciones físicas y mentales del conductor y su aptitud para conducir ^6^. El proceso de renovación del Carné Nacional de Conducción en Brasil no considera las particularidades del envejecimiento, por lo que se realiza el mismo examen en adultos y jóvenes. La única especificidad para ese grupo etario, que consta en la resolución del Consejo Nacional de Tránsito 007/98, es la reducción de cinco a tres años el período de exámenes médicos para renovar el carné de conducción después de 65 años de edad ^7^. 

Entre las inúmeras condiciones de salud en la vejez, la fragilidad física es un punto importante que debe ser investigado, por causa del aumento de su prevalencia a medida que se envejece. La fragilidad física es considerada "un síndrome médico con múltiples causas y contribuciones/determinantes, que se caracteriza por la disminución de la fuerza, resistencia y reducción de la función fisiológica que hace que aumente la vulnerabilidad del individuo y que él desarrolle mayor dependencia y/o muerte" ^8^. 

El fenotipo de fragilidad está enfocado en la perspectiva biológica del síndrome y está compuesto por cinco componentes medibles, que incluyen la reducción de la velocidad de la marcha y de la fuerza de presión manual, la pérdida de peso no intencional, bajo nivel de actividad física y el autorrelato de fatiga/agotamiento ^9^. A partir de esos cinco componentes, el anciano que presente tres o más de esas características es considerado frágil; aquel que presente uno o dos componentes, se encuentra en una fase previa al síndrome (pre-fragilidad); y aquel que no presente ninguno de los componentes citados es considerado no frágil.

Los estudios que incorporan a los ancianos en contextos diferenciados, como en clínicas de medicina de tráfico, posibilitan una evaluación más amplia de ese grupo etario y una mayor profundización en la comprensión del síndrome de la fragilidad física, subsidiando la implementación de políticas dirigidas a una conducción vehicular más segura.

Teniendo en cuenta lo anterior, el objetivo del estudio fue analizar la asociación entre la fragilidad física y los resultados de la prueba de aptitud física para la conducción vehicular en ancianos brasileños.

## Materiales y Métodos

Se trata de un estudio cuantitativo de corte transversal, durante el período de 31 de enero a 31 de julio de 2015. El estudio fue realizado en las clínicas de tráfico donde se realiza el examen de aptitud para la conducción vehicular, en la ciudad de Curitiba/PR (Brasil). Los criterios de inclusión de las clínicas fueron: estar acreditadas para realización de exámenes de aptitud física y mental y/o evaluación psicológica; tener espacio físico adecuado para la realización de las pruebas de conducción.

En el momento del delineamiento del proyecto se identificaron 54 clínicas regularmente acreditadas, que fueron incluidas para el estudio por medio de un muestreo aleatorio simple. Las clínicas fueron clasificadas con letras y números crecientes, de C1 a C54, y en esa secuencia fueron evaluadas, utilizando los criterios de inclusión. 

Considerando la inexistencia de datos referentes al número de ancianos atendidos por clínica y la distribución imparcial y equitativa de ancianos por el órgano de tráfico, se determinó realizar 35 entrevistas por clínicas, donde se incluyeron ancianos hasta el último dia de muestreo. En la última clínica, debido al término del período de muestreo, se entrevistaron 32 ancianos. 

Los criterios de inclusión del anciano fueron: tener edad igual o superior a 60 años; citados para las pruebas de conducción vehicular; ser capaz de responder a los cuestionarios del estudio. Criterios de exclusión: presentar enfermedades, problemas y síntomas físicos que por cualquier motivo, impidieran la aplicación de los cuestionarios y la realización de las pruebas de conducción.

Los ancianos fueron invitados a participar y con la firma del consentimiento informado, se colectaron los datos en tres etapas: (1) aplicación de un cuestionario estructurado; (2) pruebas de evaluación del síndrome de la fragilidad física; (3) búsqueda de los datos en el formulario de Registro Nacional de Conductores Habilitados. El cuestionario estructurado fue elaborado específicamente para el presente estudio y abordó cuestiones referentes a la dirección vehicular. Las variables de interés fueron: edad, sexo, modalidad del carné de Conducción (primera Conducción o renovación), tiempo de Conducción, características de la dirección (se dirige por la noche, en el barrio o en el centro), tipo de transmisión automotora (manual o automático), kilómetros por semana y accidentes de tránsito después de los 60 años. 

La fragilidad física se evaluó por medio de los componentes del fenotipo de Fried ^9^. La fuerza de presión manual fue medida con un dinamómetro hidráulico marca Jamar^®^. Los ancianos con valores que estuvieron en el quintil más bajo después del ajuste para sexo e índice de masa corporal, fueron considerados frágiles para este componente.

Para evaluar la velocidad da marcha, se midió el tiempo en segundos cuando el participante recorrió una línea recta, caminando un trayecto de 4.6 m en estado de reposo. Los ancianos que presentaron valores en el quintil más bajo, después del ajuste para sexo y estatura, fueron considerados frágiles para este componente.

Se verificó la pérdida de peso no intencional, según la respuesta a dos preguntas: El señor perdió peso en los últimos 12 meses? Cuántos kilogramos?. Fue considerado frágil el anciano que declaró pérdida de peso corporal &‌#8805;4.5 kg en los últimos 12 meses.

Para evaluar el componente fatiga/agotamiento, se utilizaron los ítems 7 y 20 de la Escala del *Right for Epidemiological Studies - Depression* (CES-D) (http://counsellingresource.com/quizzes/depression-testing/cesd/). Las respuestas se categorizaron de 0 a 3, de acuerdo con la frecuencia. Una respuesta 2 o 3 para cualquier una de esas preguntas categorizó el anciano como frágil para este componente.

La investigación del nivel de actividad física fue realizada por la aplicación del *Minnesota Leisure Activity Questionnaire,* validada para ancianos brasileños ^10^. Los valores que estuvieron en el quintil más bajo, después del ajuste para sexo, fueron considerados frágiles para este componente.

La metodología utilizada para la evaluación de la fragilidad física, fue propuesto por Fried *et al*, pero los puntos de corte utilizados en este estudio se definieron a partir de los resultados de la muestra. 

En el formulario de Registro Nacional de Conductores Habilitados se buscó el resultado final de la evaluación del anciano en el examen de aptitud y los valores de la fuerza de presión palmar emitidos por el médico de tráfico. El examen médico realizado incluyó un examen físico general y pruebas específicas que incluyeron una evaluación oftalmológica, otorrinolaringología, cardíacas, neurológicas y del aparato locomotor; no se hizo ninguna prueba de conducción en esta ocasión. El resultado obtenido fue presentado, conforme la resolución del Consejo Nacional de Tránsito 425 de 27 de noviembre de 2012, como "apto", "apto con restricciones", "no apto temporario" e " no apto" ^6^. En el resultado "apto con restricciones" constan las restricciones que el candidato presentó.

Los datos se organizaron en Excel^®^ 2015 y se analizaron mediante el programa *Statistical Packagefor Social Sciences* (SPSS), versión 20.0. Se utilizó la estadística descriptiva y pruebas no paramétricas (Chi-cuadrado y Cochan) para evaluar la asociación entre las variables de interés y la fragilidad física. Se consideró el *p* ≤0.05 con significancia estadística. 

El estudio fue aprobado por el Comité de Ética institucional de la Universidad Federal de Paraná (Curitiba, PR, Brasil), siendo aprobado con el registro CAAE 34689914.8.0000.0102, CEP/SD 833460. Se siguieron los principios éticos de una participación voluntaria y consentida de cada sujeto, conforme las recomendaciones contenidas en la norma vigente en Brasil, Resolución nº. 466 del Consejo Nacional de Salud, de 12 de diciembre de 2012, que considera la Declaración de Helsinki 1975 y sus revisiones posteriores. 

## Resultados 

La muestra fue constituida por 172 ancianos, con una media de edad de 67.7±6.6 años. Se incluyeron 120 (70.7%) hombres. Ningún anciano fue evaluado y categorizado como frágil; los pre-frágiles fueron 97 (56.4%) y no frágiles 75 (43.6%). El resultado final de la prueba de conducción vehicular realizado por el médico de tráfico en los ancianos mostró que el 25.0% estaban aptos, 66.6% aptos con restricción y 6.4% no aptos temporarios. 

En la Tabla 1 se observa, que la mayoría de los exámenes realizados fue para renovar el Carné Nacional de Conducción 169 (97.3%), de ellos, más del 70% tenían más de 36 años habilitados. Al asociar los datos obtenidos para el Carné Nacional de Conducción y la condición de fragilidad se observó una asociación significativa entre fragilidad y la cantidad de kilómetros conducidos semanalmente (*p*= 0.0222) y el número de accidentes (*p*= 0.0165). No hubo significancia estadística entre las características relacionadas a la cnducción vehicular (noche/día, centro/barrio y en carreteras) y la fragilidad física.

**Tabla 1:** Asociación entre la condición de fragilidad física y las características relacionadas a la dirección vehicular de los ancianos. Curitiba, PR, Brasil, 2015

Variables	Pre-frágiles (%)	No frágiles (%)	Total	*Valor p*
Tipo CNH				
Renovación	96 (98.97)	73 (97.33)	169	0.4173*
Primera vez	1 (1.03)	2 (2.67)	3	
Tiempo CNH				
≥0 a <18	7 (7.22)	8 (10.67)	15	0.5783**
≥18 a <36	19 (19.59)	13 (17.33)	32	
≥36 a <54	64 (65.98)	45 (60)	109	
≥54 a <72	7 (7.22)	9 (12)	16	
Cambio				
Manual	69(71.13)	55 (73.33)	124	0.9180*
Automático	23(23.71)	17(22.67)	40	
Manual/automático	5(5.15)	3(4)	8	
Km conducidos por semana				
≥0 a <50	16(16.49)	25(33.33)	41	0.0222**
≥50 a <250	60(61.86)	30(40)	90	
≥250 a <500	12(12.37)	13(17.33)	25	
≥500	9(9.28)	7(9.33)	16	
Accidente				
Sí	9(9.28)	1(1.33)	10	0.0165*
No	88(90.72)	74(98.67)	162	
Conducción nocturna					
Sí	72(74.23)	53(70.67)	125	0.7035**
No	25(25.77)	21(28)	46	
Conducción díurna				
Sí	96(98.97)	73(97.33)	169	0.8476*
No	1(1.03)	1(1.33)	2	
Conducción centro				
Sí	91(93.81)	64(85.33)	155	0.1030**
No	6(6.19)	10(13.33)	16	
Conducción barrio				
Sí	95(97.94)	70(93.33)	165	0.2402*
No	2(2.06)	4(5.33)	6	
Conducción carretera				
Sí	73(75.26)	63(84)	136	0.1127**
No	24(24.74)	11(14.67)	35	

*Test Cochran - test G, *p* ≤0.05

**test chi-cuadrado, *p* ≤0.05

CNH: Carné Nacional de Conducción.

En la Tabla 2 se visualiza la asociación entre la fragilidad física y los resultados obtenidos por los ancianos en el test de aptitud realizada en las clínicas de tránsito. No hubo asociación entre la clasificación del anciano, según el fenotipo de fragilidad de Fried, y los resultados finales para aptitud vehicular (*p*= 0.8934).

**Tabla 2:** Asociación entre fragilidad física y resultados finales de los exámenes de Conducción en las clínicas de tráfico. Curitiba, PR, Brasil, 2015

Resultado final del test aptitud física y mental	Pre-frágiles	No frágiles	Total	*p-value*
Apto con restricción	65(67.01)	53(70.67)	118	0.8934
Inepto temporariamente	6(6.19)	5(6.66)	11	
Apto	26(26.8)	17(22.67)	43	
Total	97(100)	75(100)	172	

Test Cochran - teste G, *p* ≤0.05.

Se presenta en la Tabla 3 la asociación estadística entre la fragilidad física y las restricciones impuestas a los candidatos considerados aptos con restricción (*p*= 0.0313). El uso de lentes correctivas fue la restricción más prevalente (n= 32; 42.66%) en los no frágiles

**Tabla 3:** Asociación entre grupos de fragilidad y el resultado apto con restricción. Curitiba-PR, Brasil, 2015

Restricciones en el Carné de Conducción^*^	Pre-frágiles	No frágiles	Total	*p-value*
Uso de lentes correctivas	26(26.8)	32(42.66)	58	0.0313
Categoría no autorizada	24(24.74)	8(10.67)	32	
Validad del CNH 2 años	2(2.06)	4(5.33)	6	
Categoría no autorizada, visión monocular y validad del CNH 2 años	1(1.03)	0(0)	1	
Uso de lentes correctivas y de prótesis auditiva	0(0)	1(1.33)	1	
Categoría no autorizada y uso de lente	11(11.34)	6(8)	17	
Categoría no autorizada y uso de prótesis auditiva	1(1.03)	0(0)	1	
Uso de lentes correctivas y validad del CNH 2 años Otras restricciones**	0(0) 1(1.03)	1(1.33) 0(0)	1 1	

^*^ El test fue realizado considerando solamente los aptos conrestricción (n= 118)

CNH: Carné Nacional de Conducción

** Otras restricciones incluyen: Prohibido dirigir encarreteras yvías de tráfico rápido, prohibido dirigir después del atardecer,validad del CNH 2 años, uso de lente y rebajamiento de categoría

Test Cochran - test G, *p* ≤0.05.

## Discusión

En las evaluaciones de los candidatos que iban a renovar el Carné Nacional de Conducción no hubo ancianos frágiles y este es un dato que difiere de la mayoría de los estudios, principalmente de los del tipo poblacional, que involucran también a los ancianos enfermos, con la capacidad funcional disminuida y ya fragilizados ^9^^,^^11^. Los ancianos que desean obtener el Carné Nacional de Conducción son independientes y no dependen de otras personas para movilizarse.

A pesar de no observarse la presencia de ancianos frágiles, la prevalencia de pre-fragilidad fue del 56.4%, resultado superior a los de estudios internacionales realizados en los Estados Unidos (40.0%) ^11^ y Taiwan (45.5%) ^12^. Porcentajes semejantes a este estudio fueron encontrados en países de América Latina, en Perú ^13^ (47.3%) y Colombia ^14^ (53.0%). En los estudios brasileños en el FIBRA (Fragilidad en Ancianos Brasileños), la pre-fragilidad varió entre 47.7% en Ivoti (RS) y 55.5% en Parnaíba (PI) ^15^. 

Es preocupante la alta frecuencia de conductores ancianos pre-frágiles, ya que es imposible predecir el tiempo de evolución de la decadencia funcional significativa, lo que podría incidir en una conducción no segura y peligrosa para la sociedad. De este modo, la identificación de los ancianos frágiles y pre-frágiles durante el examen de aptitud para la Conducción vehicular es fundamental, para para realizar una gestión específica sobre ancianos con fragilidad en los servicios de geriatría y gerontología. La gestión de la fragilidad comprende cuatro intervenciones de carácter multi-profesional: realización de ejercicios (resistencia y aeróbicos), suporte calórico y proteico, uso de vitamina D y reducción de la polifarmacia ^8^. 

Hubo asociación significativa entre el relato de accidentes de tránsito y la fragilidad física, ocurridos después de los 60 años. Este resultado indica que son necesarios estudios que desarrollen instrumentos de predicción clínica, que sean sensibles y específicos para detectar los riesgos de accidentes en conductores ancianos, principalmente en los frágiles y pre-frágiles.

Los desenlaces de los accidentes de tránsito en ancianos, pueden ser diferentes cuando se observa el grupo etario. La Comisión de Aseguradoras Australianas de Accidentes de conducción comparó dos subgrupos de conductores (edades 45-64 y 65 años y más) y se encontraron diferencias significativas respecto a los tipos de heridas. El subgrupo más joven era más susceptible a herirse en la región cervical cuando se comparó con los más viejos (30.6% y 12.1%), mientras que el subgrupo más viejo era más susceptible a daños torácicos (30.9% *versus* 18.5% del más joven). También, el estudio destacó que los más viejos pueden no tener la capacidad pulmonar para recuperarse de dichas lesiones, estando más propensos a morir de heridas torácicas ^2^.

En cuanto a los hábitos de conducción de los ancianos se observó que el 26.7% evitaron conducir por la noche y 20.4% en carreteras y/o en vías rápidas. Esta creciente auto regulación en la conducción nocturna y en carreteras se evidenció en un estudio que categorizó al conductor de acuerdo con el sexo y las edades: 55-64 años, 65-74 años, y 75 o más. El aumento de la edad fue directamente proporcional a la no conducción nocturna, con aumento de la frecuencia desde el 30.4% en los más jóvenes, 46.3% en el grupo intermedio y 56% en los mayores de 75 años. Lo mismo ocurrió con la conducción en carreteras y la edad: 18.8% de los más jóvenes y 32.9% de los más viejos evitaron la conducción. Cuando se analizó por sexo, el 34.9% de las mujeres evitaron conducir por la noche y 24.4% en carreteras, mientras que, en los hombres, ese número fue de 20.7% y 13.8%, respectivamente ^16^. 

Un estudio realizado por el *Right for Disease Control* (CDC) corroboró los resultados mencionados al mostrar que los ancianos tienden a modificar sus hábitos de conducción. El 82% de los conductores con 75 años o más dijeron limitar la conducción en la noche, o por condiciones climáticas y largas distancias. Solo el 15% de los ancianos manifestaron limitar la conducción por razones médicas y ninguno menciono que lo hacían por problemas cognitivos ^17^. 

Los conductores ancianos están conscientes de sus riesgos y utilizan acciones compensatorias, evitan situaciones de conducción peligrosas (tráfico intenso o condiciones climáticas adversas), prefieren rutas conocidas y conducción con precaución ^18^.

Hubo asociación estadística entre las restricciones establecidas para la obtención del Carné de conductor y la fragilidad física de los ancianos. Las más frecuentes fueron el uso de lentes correctivos y la categoría de conducción no autorizada. 

La obligatoriedad del uso de lentes correctivos ocurrió con menor frecuencia en los pre-frágiles comparado con los no frágiles. Se observó que diversas restricciones impuestas a los conductores no se relacionaron directamente al fenotipo de fragilidad y son fundamentales en la conducción vehicular, como la visión. 

Sobre las categorías de Conducción, llama la atención el número de ancianos con categorías de Conducción no autorizadas. Se observó que la categoría no autorizada con mayor frecuencia fue la "C" seguida por la "A". La categoría "A" comprende todos los vehículos automotores y eléctricos, de dos o tres ruedas, con o sin carro lateral, mientras que la "C" todos los vehículos automotores y eléctricos utilizados en transporte de carga, registrados con capacidad mínima de peso bruto total de 6.000 kg, cuyo peso bruto total exceda a 3.500 kg ^19^. 

Los candidatos para conducir vehículos adscritos a las categorías "C", "D" y "E", necesitan de una fuerza igual o superior a 30 kgf en cada una de las manos ^6^. De los 32 ancianos evaluados con categoría no autorizada, la mitad no obtuvo la fuerza de 30 kgf, mientras que en las clínicas de tráfico este valor fue de 31.3%, debido al intervalo de lectura del dinamómetro, que fue de 10 kgf. 

La dinamometría mínima necesaria para los candidatos a las categorías "A" e "B" debe ser igual o superior a 20 kgf ^6^. De acuerdo a la medición realizada en la evaluación de fragilidad, 8 candidatos presentaron una fuerza inferior a 20 kgf, sin embargo, ninguno fue considerado no apto para conducción vehicular. De estos, 25% fueron considerados aptos sin restricción, 50% aptos con restricción visual y 25% en categoría no autorizada, hecho este que podría resultar en una conducción insegura. Instrumentos más precisos pueden ser útiles para informarle al candidato que la fuerza de presión manual está próxima a la mínima necesaria y orientar las actividades para que estos ancianos ganen fuerza muscular.

La existencia de la relación entre la fragilidad física y los aptos con restricción evidencia la necesidad de una gestión de la fragilidad efectiva, que se debe iniciar, por lo menos, durante la evaluación de la aptitud, para poder mantener las habilidades específicas de conducción y desempeño de conducción ideal. Si se les restringe conducir a los ancianos les puede afectar negativamente la calidad de vida, lo que puede acelerar incluso, la senescencia ^20^. A pesar de no haber asociación entre la fragilidad física y el resultado de conducción vehicular, se observó asociación entre la fragilidad y la restricción impuesta en el Carné de Conducción. 

La ausencia de un puntaje para la velocidad de la marcha, nivel de actividad física y la fuerza de presión palmar, presentes en el fenotipo de fragilidad, como también la presencia de componentes de auto reporte como la fatiga/agotamiento, nivel de actividad física y pérdida de peso no intencional pueden influir en la clasificación de los ancianos.

Otras limitaciones se refieren a la posibilidad de omisión de datos por parte del anciano, durante la evaluación de la fragilidad física y al diseño transversal del estudio, que no proporciona el entendimiento de algunas relaciones de causa y efecto entre las variables.

## Conclusiones

La fragilidad física estuvo asociada a las restricciones relacionadas con la conducción. Sin embargo, no se observó una asociación entre fragilidad física y el resultado final la conducción de vehículos, esto también se observó en una parte de los ancianos pre frágiles. Esta información demuestra la importancia de realizar intervenciones en esta fase de la vida, para tener una mayor posibilidad y efectividad de la interrupción y/o reversión de esta condición y consecuentemente mantener una conducción segura por un mayor tiempo.
